# Time-to-Surgery and Short-Term Outcomes of Trimalleolar Ankle Fracture During the COVID-19 Pandemic

**DOI:** 10.7759/cureus.44478

**Published:** 2023-08-31

**Authors:** Gabriel B Burdick, Rami S Beydoun, Kerri L Bell, Bushra Fathima, Alexander D Pietroski, Jonathan R Warren, Trevor D Wolterink, Johnny K Kasto, Ryan Y Sanii, Stephanie Muh

**Affiliations:** 1 Department of Orthopaedic Surgery, University of Southern California, Los Angeles, USA; 2 Department of Orthopaedic Surgery, Beaumont Hospital, Royal Oak, USA; 3 Department of Orthopaedic Surgery, Henry Ford Health System, Detroit, USA; 4 Department of Neurosurgery, Yale School of Medicine, New Haven, USA; 5 Department of Radiology, Beaumont Hospital, Royal Oak, USA; 6 Department of Orthopaedic Surgery, University of Missouri Kansas City, Kansas City, USA; 7 Department of Orthopaedic Surgery, Wayne State University School of Medicine, Detroit, USA

**Keywords:** pandemic response, orthopaedic surgery, coronavirus disease 2019 (covid-19), trimalleolar ankle fracture, trauma

## Abstract

Introduction

During the coronavirus disease 2019 (COVID-19) pandemic, a rapid and significant transformation in patient management occurred across the healthcare system in order to mitigate the spread of the disease and address resource constraints. Numerous surgical cases were either postponed or canceled, permitting only the most critical and emergent cases to proceed. The impact of these modifications on patient outcomes remains uncertain. The purpose of this study was to compare time-to-surgery and outcomes of open reduction and internal fixation for trimalleolar ankle fractures during the pandemic to a pre-pandemic group. We hypothesized that the pandemic group would have a prolonged time-to-surgery and worse outcomes compared to the pre-pandemic cohort.

Materials and methods

This retrospective cohort study was conducted within a single healthcare system, examining the treatment of trimalleolar ankle fractures during two distinct periods: April to July 2020 (COVID-19 group) and January to December 2018 (2018 group). Cases were identified using Current Procedural Terminology code 27822. Information on demographics, fracture characteristics, and outcomes was obtained through chart review. Outcomes analyzed included time-to-surgery, mean visual analog scale scores, ankle strength and range of motion, and complications.

Results

COVID-19 and 2018 groups consisted of 32 and 100 patients, respectively. No significant difference was observed in group demographics and comorbidities (p > 0.05). Fracture characteristics were similar between groups apart from tibiofibular syndesmosis injury, 62.5% (20/32) in COVID-19 vs 42.0% (42/100) in 2018 (p = 0.03). Time-to-surgery was not significantly different between the two groups (8.84 ± 6.78 days in COVID-19 vs 8.61 ± 6.02 days in 2018, p = 0.85). Mean visual analog scale scores, ankle strength, and ankle range of motion in plantarflexion were not significantly different between the two groups at three and six months postoperatively (p > 0.05). Dorsiflexion was significantly higher in the COVID-19 group at three months (p = 0.03), but not six months (p = 0.94) postoperatively. No significant difference in postoperative complication was seen between groups, 25.0% (8/32) COVID-19 group compared to 15.0% (15/100) 2018 group (p = 0.11).

Conclusions

Patients who underwent surgery during the early months of the COVID-19 pandemic did not experience prolonged time-to-surgery and had similar outcomes compared to patients treated prior to the pandemic.

## Introduction

Trimalleolar ankle fractures are fractures involving all three malleoli of the ankle. It is a relatively uncommon but severe type of ankle fracture, requiring prompt surgery in most cases [1]. Delayed surgery has been associated with increased risk for complications and worse functional outcomes related to these complications [2]. Worse outcomes have also been linked to patient comorbidities, open fractures, fracture dislocations, and the size of the posterior fracture segment [3-6].

The World Health Organization’s 2020 declaration of the COVID-19 pandemic resulted in drastic worldwide health system reorganization measures to stem the spread of the disease and conserve limited healthcare resources [7]. As part of this response, elective orthopedic surgical procedures were postponed across the United States [8]. However, many conditions treated by orthopedic surgeons continued to be prioritized according to published guidelines [9-11]. Despite best efforts to triage orthopedic patients requiring urgent and emergent surgery, suboptimal conditions created by widespread disruption to the healthcare system may have broadly impacted the outcomes of these surgeries.

Therefore, the aim of this study was to determine if the COVID-19 pandemic impacted surgical treatment of trimalleolar ankle fractures at a large, academic health system by evaluating (1) time-to-surgery, (2) complication rates, and (3) functional outcomes in patients who underwent open reduction and internal fixation (ORIF). We hypothesized that patients who underwent ORIF of trimalleolar ankle fractures during the COVID-19 pandemic experienced prolonged time-to-surgery, higher complication rates, and poorer functional outcomes when compared to pre-pandemic patients. We undertook a retrospective cohort study to compare time-to-surgery and outcomes in patients who had undergone surgery prior to the COVID-19 pandemic and during the COVID-19 pandemic.

## Materials and methods

This was a retrospective cohort study consisting of two cohorts created according to their date of surgery. The COVID-19 pandemic cohort consisted of patients who underwent surgery at the immediate start of the pandemic from April 1 to July 31 of 2020 (COVID-19 group). The pre-pandemic cohort consisted of patients who underwent surgery prior to the pandemic from January 1 to December 31 of 2018 (2018 group). Patients ≥18 years of age who underwent surgical management for trimalleolar ankle fracture at our institution during the study period were eligible for inclusion. Patients without follow-up at three to six months postoperatively were excluded from the study.

Following expedited institutional review board approval by Henry Ford Health System Institutional Review Board (approval 13963-01), patients who underwent ORIF for trimalleolar ankle fracture were identified by querying a billing record database for Current Procedural Terminology code 27822. A manual retrospective chart review was conducted by research assistants to obtain information from the medical charts of patients who met inclusion criteria for this study. Age, sex, race, body mass index, zip code, smoking history, and a prior diagnosis of type 2 diabetes mellitus were recorded. Zip code was cross-referenced with a United States Census Bureau website to determine median household income [12]. Preoperative imaging reports and provider notes were reviewed by resident physicians to determine the following ankle fracture characteristics: open vs closed, comminution of the posterior malleolus, and tibiofibular syndesmosis injury. Time-to-surgery was calculated as the number of days from date of injury to the date of surgery. Provider notes were reviewed up to one year postoperatively to determine whether a complication occurred after surgery. Visual analog scale (VAS) pain scores, ankle strength, and active ankle range of motion (ROM) measurements were collected from the surgeon’s encounter note for the three- and six-month postoperative clinic visits. VAS pain score was measured on a scale of 0-10. Ankle strength was measured on a scale of 0-5, and ROM was measured in degrees of active motion.

Statistical analysis was performed using SAS 9.4 (SAS Institute Inc, Cary, NC, USA) with significance set at p-value < 0.05. Sex, race, marital status, and complication rate were reported as frequency and percentages and analyzed using chi-square test. Age, median income, and time-to-surgery were reported as mean ± standard deviation and analyzed using two-sample T-test. Ankle strength, ankle ROM, and VAS score at three and six months postoperatively were analyzed using the Wilcoxon rank-sum test.

## Results

Thirty-two patients (11 female and 21 male) underwent ORIF for trimalleolar ankle fracture between April and July 2020 and met inclusion criteria for this study, compared to 100 patients (73 female and 27 male) in 2018. The groups were not significantly different with regards to age, sex, race, income, marital status, smoking status, or incidence of diabetes (p > 0.05). Demographic information is presented in Table 1.

**Table 1:** Patient demographics Abbreviations: SD, standard deviation; US, United States

		2018 Group (n=100)	COVID-19 Group (n=32)	P-value
Age (years), mean ± SD		56.9 ± 18.3	57.2 ± 15.1	0.93
Sex	Male	27 (27.0%)	11 (34.4%)	0.42
	Female	73 (73.0%)	21 (65.6%)	
Race	White	73 (73.0%)	23 (71.9%)	0.12
	Black	20 (20.0%)	4 (12.5%)	
	Other	7 (7.0%)	5 (15.6%)	
Median income (US dollars), mean ± SD		$61,041 ± $26,217	$58,749 ± $27,555	0.67
Marital status	Single	33 (33.0%)	8 (25.0%)	0.43
	Married	42 (42.0%)	17 (53.1%)	
	Other	25 (25.0%)	7 (21.9%)	
Type 2 diabetes mellitus		21 (21.0%)	8 (25.0%)	0.58
Current or former smoker		49 (49.0%)	17 (53.1%)	0.64

The most common mechanism of injury in both groups was fall, 84.3% (27/32) in the COVID-19 group vs 84.0% in the 2018 group (84/100). Mechanism of injury did not significantly differ between the two groups (p = 0.74). Comparison of ankle fracture characteristics revealed similar frequencies of open fractures (6.3% in COVID-19 vs 12.0% in 2018, p = 0.32) and comminution of the posterior malleolus (12.5% in COVID-19 vs 7.0% in 2018, p = 0.23) between the groups, but a higher frequency of tibiofibular syndesmosis injury in the COVID-19 group (62.5% in COVID-19 vs 42.0% in 2018, p = 0.03). There was no significant difference between the COVID-19 group and the 2018 group when comparing the rates of additional fractures other than ankle fracture, 21.9% (7/32) and 18.0% (18/100), respectively (p = 0.57). Similarly, there was no significant difference in external fixation prior to definitive ORIF between the COVID-19 group and the 2018 group, 15.6% (5/32) compared to 12.0% (12/100), respectively (p = 0.53). Injury and fracture characteristics are presented in Table 2.

**Table 2:** Injury and fracture characteristics Abbreviation: ORIF, open reduction and internal fixation

		2018 Group (n=100)	COVID-19 Group (n=32)	P-Value
Mechanism of injury	Fall	84 (84.0%)	27 (84.3%)	0.74
	Motor vehicle accident	5 (5.0%)	3 (9.4%)	
	Other	11 (11.0%)	2 (6.3%)	
Fracture characteristics	Open	12 (12.0%)	2 (6.3%)	0.32
	Tibiofibular syndesmosis injury	42 (42.0%)	20 (62.5%)	0.03
	Comminution of posterior malleolus	7 (7.0%)	4 (12.5%)	0.23
	Additional fracture(s)	18 (18.0%)	7 (21.9%)	0.57
	External fixation prior to ORIF	12 (12.0%)	5 (15.6%)	0.53

Time-to-surgery was similar between groups, with an average of 8.84 ± 6.78 days for the COVID-19 group, compared to 8.61 ± 6.02 days in the 2018 group (p = 0.85). There was a 10% difference in complication rate between the two groups, with 25.0% (8/32) of patients in the COVID-19 group experiencing one or more complications related to their surgery up to one year postoperatively, compared to 15.0% (15/100) in the 2018 group. However, the difference in complication rate between the two groups was not statistically significant (p = 0.19). Postoperative complications and their frequencies are detailed in Table 3.

**Table 3:** Postoperative complications

	2018 Group (n=100)	COVID-19 Group (n=32)
Elective hardware removal due to pain or stiffness	8 (8.0%)	3 (9.4%)
Broken or loosened hardware	3 (3.0%)	3 (9.4%)
Superficial wound complication	10 (10.0%)	2 (6.3%)
Deep tissue infection	2 (2.0%)	0 (0.0%)
Traumatic refracture	0 (0.0%)	2 (6.3%)
Fracture nonunion or delayed union	2 (2.0%)	0 (0.0%)
Ankle malalignment	0 (0%)	1 (3.1%)

Functional outcomes examined by this study were postoperative pain levels, ankle strength, and ankle ROM at three and six months postoperatively. Mean VAS pain score was not significantly different between the groups at three months (2.85 in COVID-19 vs 3.32 in 2018, p = 0.42) and six months (2.14 in COVID-19 vs 4.43 in 2018, p = 0.07) postoperatively. Strength of ankle dorsiflexion, plantarflexion, eversion, and inversion were not significantly different between the groups at three months (p = 0.73, 0.72, 0.84, and 0.70, respectively) and six months (p = 0.69, 0.53, 0.69, and 0.69, respectively) postoperatively. ROM in ankle dorsiflexion was significantly higher in the COVID-19 group at three months (9.50 in COVID-19 vs 9.06 in 2018, p < 0.05), but not six months postoperatively (p = 0.94). ROM in plantarflexion was not significantly different between the groups at three months (p = 0.96) and six months (p = 0.84) postoperatively.

Considering the well-documented effect of seasonality on fracture frequencies and characteristics [13,14], this study aimed to identify and eliminate date of service as a potential confounding factor. Therefore, the COVID-19 group was compared to a subset of patients in the 2018 group who underwent surgery between April and July of 2018. This subgroup analysis revealed a higher proportion of patients in the COVID-19 group with tibiofibular syndesmosis injury compared to the 2018 subgroup (62.5% in COVID-19 vs 38.9% in 2018 subgroup, p = 0.01) and a significant difference between the groups with regards to race (p = 0.001). There were no other significant differences between the groups regarding patient or fracture characteristics, time-to-surgery, complications, or functional outcomes.

## Discussion

The purpose of this study was to determine the impact of the COVID-19 pandemic on surgical timing and outcomes of ORIF for trimalleolar fractures at a large, academic health system. The study compared a cohort treated during the early months of the COVID-19 pandemic (COVID-19 group) to an equitable pre-pandemic cohort (2018 group). The number of patients in the COVID-19 group compared to the 2018 group was reduced possibly as a result of behavior or activity changes during the pandemic [15,16]. We found that the COVID-19 group did not experience inferior functional outcomes. Specifically, VAS pain scores, ankle ROM, and ankle strength were similar between the groups at three and six months postoperatively. Although surgery-related complications were higher in the COVID-19-affected cohort (25% vs 15% in 2018), this difference was not statistically significant (p = 0.19).

Demographically, we did not find any major differences between the 2018 and COVID-19 groups. Patients were similar regarding age, sex, race, income, marital status, smoking status, and incidence of diabetes. Additionally, there was no significant difference in mechanism of injury or the majority of ankle fracture characteristics between groups. However, the COVID-19 cohort had a higher frequency of tibiofibular syndesmosis injury in comparison to the 2018 cohort.

An additional key finding from our study was that surgery for trimalleolar ankle fracture was not significantly delayed during the early months of the COVID-19 pandemic. The COVID-19 and 2018 groups underwent surgery 8.84 ± 6.78 and 8.61 ± 6.02 days from their date of injury, respectively (p = 0.85). Currently, there is no consensus and conflicting evidence in the scientific literature regarding optimal time to surgical fixation [17-19]. A retrospective study by Naumann et al. of 1,011 patients with closed ankle fractures who underwent ORIF found no significant difference in postoperative length of stay or complications after adjusting for patient and fracture characteristics in patients who underwent surgery <eight hours, eight hours to six days, or greater than six days after injury [20]. However, other studies have shown that the incidence of wound complications and infection increases with delay. For example, a retrospective study of 85 patients by Saithna et al. undergoing ORIF for closed ankle fractures found that the incidence of infection was significantly higher in patients who underwent surgery after six days (3.6 vs. 20.7%, p = 0.010) [21]. Though system-wide efforts were made to reduce the number of inpatient admissions, as most ankle fractures are treated as outpatient cases, this reduction of inpatient admissions did not significantly impact the management of trimalleolar fracture care during the pandemic at our institution. In fact, our institution was able to effectively utilize affiliated surgical centers to perform outpatient surgeries without creating further burden on the overstressed inpatient hospitals. The only additional measures our institution took was screening patients for COVID-19 and isolating any patients with COVID-19 or suspected COVID-19 symptoms. This consistency of care between the two cohorts may be why we did not see significant differences in the outcomes between the two groups.

Additionally, to our knowledge, no study has previously examined time-to-surgery for trimalleolar ankle fractures within United States hospital systems during the pandemic. A study by Wong and Cheung analyzed 122 outpatient clinics in Hong Kong and demonstrated that patients did not endure longer wait times for emergency operations or consultations [22]. While there was a deficit in the time to surgery studies, there were a few studies that analyzed the effect of a positive COVID-19 diagnosis on ankle fracture treatment. A retrospective study of ankle fractures during the pandemic found that COVID-19-positive patients who had ankle fracture surgery had significantly longer times to treatment and longer hospital length of stay than the COVID-19-negative group [23]. A retrospective study by Mercier et al. found that COVID-19-positive patients were at an increased risk for perioperative complications following ORIF. While there was a significant increased risk for any adverse event, serious adverse events, or minor adverse events, there was also found to be an increased risk for sepsis, pneumonia, and acute kidney injury in the COVID-19-positive cohort [23]. In the study done at our institution, none of the patients treated for a trimalleolar ankle fracture had COVID-19, further supporting the consistency in outcomes between our two cohorts.

This study has several limitations, the first being that this was a retrospective study, with all the inherent limitations related to the quality or process of record-keeping. Additionally, retrospective studies are susceptible to biases, such as selection bias [24]. We worked to mitigate such biases by matching the COVID-19 and 2018 group for age, sex, race, income, and marital status. However, the only comorbidities and risk factors our study considered were smoking and diabetes. A study by Jupiter et al. found that other factors such as myocardial infarction, history of a cerebrovascular accident, systemic lupus erythematosus, male sex, and increased age preoperatively were associated with postoperative complications including infection, pulmonary embolism, or repeat surgery [3]. Furthermore, we recognize that the sample size of the study is limited with 32 patients in the COVID-19 cohort compared with 100 patients in the 2018 cohort, which holds implications regarding the power of the study. Additionally, ORIF was performed by multiple surgeons, which introduces the possibility of differences in surgical technique and proficiency.

## Conclusions

This study concludes that the COVID-19 pandemic did not have a significant impact on trimalleolar fracture outcomes at our institution. Time-to-surgery was not significantly different in the pandemic group compared to the 2018 group. There were non-significant differences in VAS scores, ankle strength, and ankle ROM at three and six months postoperatively and complications up to one year postoperatively.
